# Testing the controllability of contextual cuing of visual search

**DOI:** 10.1038/srep39645

**Published:** 2017-01-03

**Authors:** David Luque, Miguel A. Vadillo, Francisco J. Lopez, Rafael Alonso, David R. Shanks

**Affiliations:** 1 School of Psychology, UNSW Australia, Sydney NSW 2052, Australia; 2 Department of Primary Care and Public Health Sciences, King’s College London SE1 1UL, United Kingdom; 3 Departamento de Psicología Básica, Universidad Autónoma de Madrid, 28049, Spain; 4 Departamento de Psicología Básica, Instituto de Investigación Biomédica de Málaga (IBIMA), Universidad de Málaga, Málaga 29071, Spain; 5 Division of Psychology and Language Sciences, University College London, London WC1H 0AP, United Kingdom

## Abstract

Locating a target among distractors improves when the configuration of distractors consistently cues the target’s location across search trials, an effect called c*ontextual cuing* of visual search (CC). The important issue of whether CC is automatic has previously been studied by asking whether it can occur implicitly (outside awareness). Here we ask the novel question: is CC of visual search controllable? In 3 experiments participants were exposed to a standard CC procedure during Phase 1. In Phase 2, they localized a new target, embedded in configurations (including the previous target) repeated from Phase 1. Despite robust contextual cuing, congruency effects – which would imply the orientation of attention towards the old target in repeated configurations – were found in none of the experiments. The results suggest that top-down control can be exerted over contextually-guided visual search.

Real life scenes include multiple and complex elements. In order to achieve our ongoing goals, we need to know not only what to search for, but also *where* to look. Elements forming part of the background context can guide visual search when they reliably predict the location the object of interest. When driving along a cluttered but familiar road, for example, buildings, trees and so on can guide our attention and facilitate detection of a traffic signal^1^. In these cases, c*ontextual cuing* of visual search (hereafter CC) assists us in quickly scanning a scene in order to guide behavior.

CC has been extensively investigated using a task originally developed by Chun and Jiang^2^. In this task participants respond as rapidly as possible to the orientation of a target stimulus (such as the letter T) presented among a set of distractor stimuli (e.g., the letter L). Some of the distractor-target configurations are repeated during the course of the experiment, and therefore participants can speed up their visual search by learning these configurations and using them to guide attention towards the target’s location. For other configurations the distractors are distributed at random on every trial. Response times (RTs) to detect the target stimulus in repeated configurations become faster across training compared with RTs in the random configurations - this facilitation effect is what is called CC (for a review, see ref. 3).

CC may be observed even when participants are not able to identify the learned distractor-target configurations in a post-learning recognition test^2^. On the basis of such results, it has been proposed that CC is produced by automatic implicit (unconscious) learning of the repeated configurations (refs 4 and 5; but see ref. 6). However, ‘automaticity’ is a complex concept that is best thought of as a collection of distinct features than as a unitary process. Among the most important features of automatic processing, besides the fact of being unconscious, are uncontrollability and independence from the deployment of cognitive resources^7^. Importantly, these features have to be explored independently in order to properly characterize the nature of a process (e.g. ref. 8). Surprisingly, although a large body of research has explored whether CC operates unconsciously^6^, and whether it depends on working memory resources^9^, there is a lack of research regarding its controllability. The current study aimed to fill this gap and asks the novel question of whether contextual cuing of visual search is controllable.

A psychological process is said to be *uncontrollable* if it affects behavior even when the performer voluntarily attempts to avoid its influence because it is counterproductive for the ongoing task goals^10^,^11^. Thus, in the current study we followed an *interference* logic in order to assess whether CC is uncontrollable. A well-known example of this strategy is the *Stroop* task, in which naming the color in which a word is printed takes more time when the word names a different color (an *incongruent* trial, e.g., the word ‘red’ printed in green) compared to when the color of the word matches its name (*congruent* trials, e.g., the word ‘red’ printed in red)^12^.

To implement this strategy, a two-phase contextual cuing paradigm was used in the current experiments. In Phase 1, participants completed a standard CC task to establish a set of displays in which distractor configurations predicted the location of a target letter T. Phase 2 was similar to Phase 1, with the exception that a new target stimulus (the letter Y) was added. Participants were instructed to respond to the orientation of the new target and ignore the previous target stimulus — and of course all the distractors. Importantly, in half of the trials both targets were orientated in the same direction—*congruent* trials— and for the remaining trials they were orientated in opposite directions—*incongruent* trials. Participants were explicitly instructed about the location where the new target stimulus would appear in the visual scene; therefore, any interference from the Phase 1 target must be attributed to uncontrollable visual orientation towards the previously relevant target.

If the contextual cuing effect observed in Phase 1 exerts an uncontrollable influence on Phase 2 performance, then we should expect a larger congruency effect in repeated patterns than in random patterns, because repeated configurations have the capacity to prime search towards the location of the Phase 1 target via CC, thus yielding facilitation when the 2 targets are congruent or interference when they are incongruent (see Fig. 1). Thus, RTs to the Phase 2 target (Y) should be faster on congruent than incongruent trials in repeated patterns where visual search is effectively cued by training in Phase 1.

## Results

### Experiment 1

**Figure 1:**
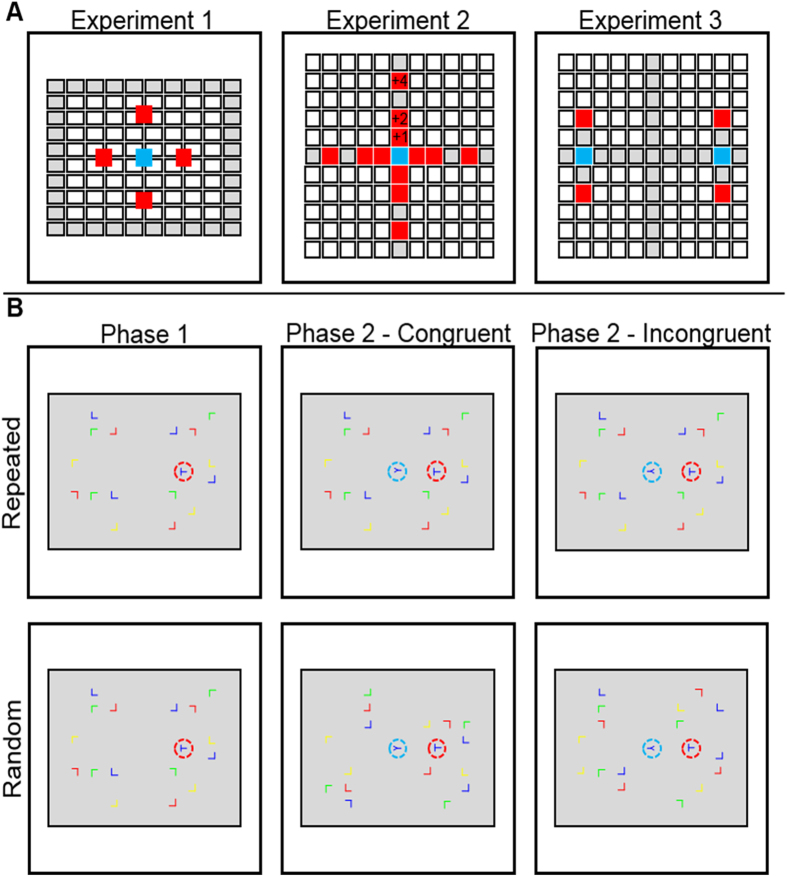
Task used in Experiments 1–3. (**A**) Locations of the invisible grid where stimuli could be presented. Red squares denote locations reserved for T-shaped Phase 1 targets, while blue squares denote locations reserved for Phase 2 Y-shaped targets. Distractors could appear in any other location, except the ones colored in grey, which were always empty. The +1, +2 and +4 symbols indicate the three eccentricity conditions included in Experiment 2. Note that the boxes were not marked on the display itself. (**B**) Examples of repeated and random trials (upper and lower rows respectively) from Experiment 1. First column: Trials from the first phase (i.e., a standard CC paradigm). Second column: Congruent trials from the second phase. Third column: Incongruent trials also from the second phase. The red circle highlights the position of the Phase 1 target and the blue circle the position of the Phase 2 target. These circles were not presented during the actual experiments.

In Experiment 1 the target stimulus for the second phase (a rotated letter Y) always appeared in the center of screen, while the stimulus which played the role of the target during Phase 1 (a rotated letter T) could appear either at the left, right, up, or down from the Y target. The distance between targets was kept constant (Methods and Fig. 1).

**Figure 2:**
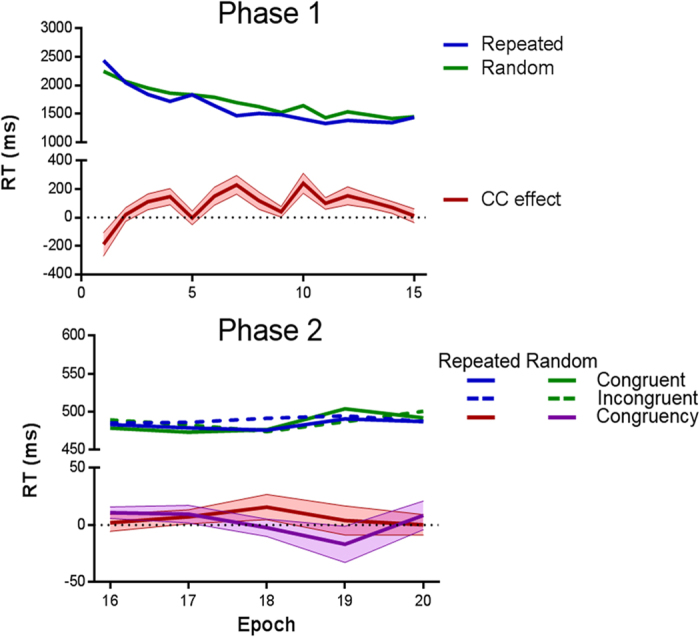
Experiment 1 results. Top panel: Data from Phase 1. ‘CC effect’ refers to the magnitude of the Contextual Cuing effect (random minus repeated RTs). Bottom panel: Data from Phase 2. ‘Congruency’ refers to the incongruent minus congruent subtraction. Semi-transparent areas represent SEM.

Figure 2 shows RTs to the target stimulus across epochs for Phases 1 and 2. Regarding Phase 1, a 2 (configuration: repeated vs. random) ×15 (epoch) ANOVA yielded a main effect of configuration, *F*(1,29) = 16.19, *P* < 0.001, a main effect of epoch, *F*(4.97,144.25) = 38.02, *P* < 0.001, and a significant configuration × epoch interaction, *F*(6.61,191.76) = 3.75, *p* = 0.001. As can be observed in Fig. 2, these results are a consequence of faster RTs for repeated than for random configurations, confirming the robust development of CC.

RTs in Phase 2 were analyzed with a repeated-measures ANOVA with factors of configuration, congruency, and epoch. This revealed a main effect of congruency, *F*(1,29) = 5.86, *P* = 0.022, with faster RTs when the T and Y stimuli were oriented in the same direction. None of the other effects was significant (smallest *P* > 0.2). Crucially, there was no evidence of uncontrollable contextually-guided attention towards the Phase 1 target: The congruency effect was not significantly larger for repeated (*M* = 5.89 ms) than for random (*M* = 1.97 ms) configurations (Fig. 2).

RTs were faster for repeated than random displays during Phase 1, demonstrating CC. During Phase 2 this learned attentional orientation had little (if any) impact on responding to the Y target. Importantly, the second phase data analysis revealed null results regarding the configuration × congruency interaction. To quantify the extent to which the data favor this null hypothesis, we conducted a Bayesian analysis on the second phase RTs. In order to calculate a Bayes Factor (BF) against the specific 2 (configuration) ×2 (congruency) interaction, we collapsed RTs across blocks, and then calculated a new dependent *congruency* variable (incongruent minus congruent RTs). A Bayesian paired *t*-test was performed on this new variable with *configuration* as factor. In this analysis (as in the equivalent analysis in the following experiments) we tested the null hypothesis (i.e., the same effect of congruency for repeated and random configurations) against the H1 of a larger congruency effect for repeated than random configurations. A BF_01_ larger than 3 is usually considered to reflect substantial support for the null hypothesis and a value larger than 10 strong support. Conversely, values lower than 1/3 are considered substantial evidence and values lower than 1/10 strong support for the alternative hypothesis^13^. The BF_01_ in favor of the null hypothesis was 2.67. The same analysis was repeated for the subset of participants who showed numerical CC (i.e., faster RTs for repeated than random configurations during Phase 1) (N = 25); data from this subset of participants did not support the alternative hipothesis either, BF_01_ = 2.35. In addition, we repeated the analysis with the subset of participants who showed numerical CC in the last epoch of Phase 1 (N = 15), yielding a result even more favorable for the null hypothesis, BF_01_ = 3.70.

Finally, we explored the correlation between the magnitudes of CC and of the selective congruency effect. CC was computed as the difference in RTs to repeated and random patterns during Phase 1. The selective congruency effect is defined as the difference between (a) the difference of RT in incongruent and congruent repeated trials and (b) the difference of RT in incongruent and congruent random trials. Because we had a clear prediction of the direction of the correlation (positive), the statistical test for this contrast was 1-tailed. This analysis was not significant, *r*(28) = 0.12, *P* > 0.2.

CC is thought to be resistant to extinction^14^, but nevertheless some degree of weakening might be expected during Phase 2. Although the distractor configurations continued to predict the location of the T stimulus, this was no longer the search target during Phase 2. In order to further explore the relationship between the magnitude of CC (first phase) and the selective congruency effect (second phase), we computed the correlation between these two variables but only taking into consideration the first epoch of Phase 2. This analysis was not significant in a 1-tailed test; indeed, the direction of the relationship was negative, *r*(28) = −0.42.

### Experiment 2

In Experiment 2 we explored three different between-target eccentricities, searching for the optimal display conditions for obtaining a configuration × congruency interaction. We anticipated a larger congruency effect overall when the T and Y were closer together in Phase 2. Following the same aim, we increased the number of participants in Experiment 2 as compared with Experiment 1.

**Figure 3:**
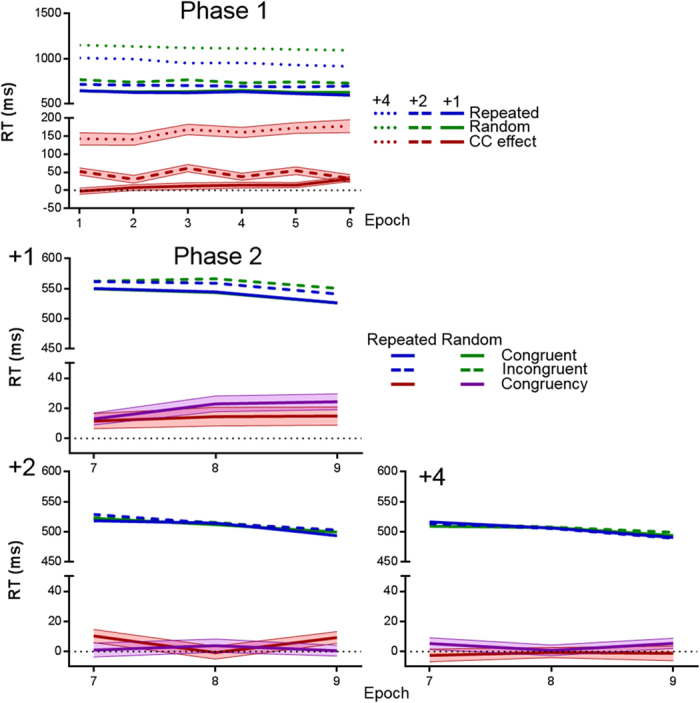
Experiment 2 results. Top panel: Data from Phase 1. ‘CC effect’ refers to the magnitude of the contextual cuing effect (random minus repeated RT). Middle and lower panels: Data from Phase 2. ‘+4’, ‘+2’, and ‘+1’ denote the three experimental conditions which differed in terms of between-target eccentricity. ‘Congruency’ refers to the incongruent minus congruent subtraction. Semi-transparent areas illustrate SEM.

Figure 3 shows RTs to the target stimuli. Random displays were introduced only in Phase 1B, therefore Phases 1B and 2 were considered for RT analysis. Regarding Phase 1B, a 2 (configuration: repeated vs. random) ×3 (eccentricity: +1, +2, +4) ×6 (epoch) ANOVA yielded a main effect of configuration, *F*(1,105) = 422.36, *P* < 0.001, a main effect of eccentricity, *F*(1.18,124.18) = 1143.64, *P* < 0.001, a main effect of epoch, *F*(3.32, 348.16) = 9.47, *P* < 0.001, 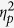 = 0.08, a significant configuration × eccentricity interaction, *F*(1.65, 172.87) = 200.62, *P* < 0.001, and a significant eccentricity × epoch interaction, *F*(7.69, 807.28) = 4.94, *P* < 0.001. The configuration × epoch and configuration × eccentricity × epoch interactions were not significant [*P* = 0.082 and *P* = 0.390, respectively.] As can be observed in Fig. 3, the results can be summarized as revealing faster RTs for repeated than for random configurations (i.e., CC), slower RTs with increasing eccentricity, and a larger CC effect for those conditions with larger eccentricity: The CC effect was larger in the +4 condition than in the +2, *t*(105) = 5.54, 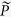 < 0.001, and the +1 condition, *t*(105) = 18.11, 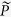 < 0.001, and larger in the +2 than in the +1 condition, *t*(105) = 12.88, 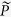 < 0.001 (Bonferroni-Holm corrected *P*-values).

Regarding Phase 2, a 2 (configuration) ×2 (congruency) ×3 (eccentricity) ×3 (epoch) ANOVA revealed main effects of congruency, *F*(1, 105) = 34.23, *P* < 0.001, eccentricity, *F*(1.21, 126.90) = 156.27, *P* < 0.001, 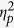
= 0.60, and epoch, *F*(1.48, 155.43) = 12.80, *P* < 0.001, and a congruency × eccentricity interaction, *F*(2, 210) = 22.51, *P* < 0.001. All remaining effects were nonsignificant; the closest to significance was the configuration × congruency × eccentricity interaction, *P* = 0.123. Thus, as in Experiment 1, configuration (repeated or random) did not affect the congruency effect. On the other hand, as expected the eccentricity of the T stimulus influenced the congruency effect, with a larger congruency effect when the T and Y stimuli were closer to each other (Fig. 3). Indeed, the magnitude of congruency effect (i.e., congruent minus incongruent trials) was significantly different from zero only at the +1 and +2 eccentricity conditions (+1 *M* = 16.87 ms, *t*(105) = 7.21, *P* < 0.001; +2 *M* = 4.03 ms, *t*(105) = 2.17, *P* = 0.032; +3* M* = 1.14 ms, *t*(105) < 1).

Although the congruency effect was reliably larger when the T was close to the Y, it was not significantly larger for repeated than for random configurations at the +1 eccentricity (repeated *M* = 13.11 ms, random *M* = 20.08 ms), the +2 eccentricity (repeated *M* = 6.34 ms, random *M* = 1.47 ms), or the +4 eccentricity (repeated *M* = −2.19 ms, random *M* = 3.60 ms).

We conducted Bayesian *t*-tests assessing whether the congruency effect was larger in repeated patterns at each level of the eccentricity factor. In the +1 condition the BF_01_ against a larger effect of congruency in repeated than random displays was 20.29, in the +2 condition BF_01_ = 2.19, and in the +4 condition BF_01_ = 23.33. Thus, the only condition in which the BF does not clearly support the null hypothesis was condition +2, but even in this condition the BF favors the null over the alternative hypothesis. The same analyses for the subset of participants who showed numerical CC during Phase 1 showed similar results, +1 BF_01_ = 20.42, N = 68; +2 BF_01_ = 2.46, N = 88; +4 BF_01_ = 21.91, N = 104. These results did not change for the subset of participants with numerical CC just in the last epoch of Phase 1, +1 BF_01_ = 15.46, N = 71; +2 BF_01_ = 3.95, N = 69; +4 BF_01_ = 23.50, N=91.

As in Experiment 1, we explored a possible relationship between the magnitude of CC in the first phase and the selective congruency effect during the second phase. We performed three correlations, one for each level of eccentricity, between the overall magnitude of CC during Phase 1B and the selective congruency effect computed across all epochs from Phase 2. These correlations were 2-tailed, because the results from Experiment 1 indicate that these correlations can take either positive or negative values. Results showed no reliable correlations, regardless of the eccentricity condition, +1: *r*(104) = −0.21, 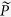 = 0.081; +2: *r*(104) = 0.05, 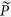 = 0.634; +4: *r*(104) = −0.16, 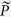 = 0.188. The same analysis, but using only the first epoch from the second phase for computing the selective congruency effect, revealed a similar pattern, +1: *r*(104) = −0.13, 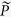 = 0.332; +2: *r*(104) = 0.23, 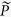 = 0.057; +4: *r*(104) = −0.11, 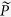 = 0.277.

### Experiment 3

A difference between the Phase 2 procedure in Experiments 1 and 2 and most typical CC experiments is that in the latter participants have to direct their attention away from the center of the screen in order to find the target. Therefore, it is possible that the uncontrollable orientation (if any) produced during a CC task may require that participants engage in active visual search. A fairer condition to test the uncontrollability hypothesis for CC may therefore be one in which the participants have to search for the target, as they actually do in a CC experiment (or in Phase 1 of our experiments). In Experiment 3, which was preregistered (https://osf.io/e3gb5/), the Y target was located in one of two possible positions during Phase 2 (left or right); hence, participants had to search for it (see Fig. 1A). Participants were informed about the two possible locations of the Y target at the beginning of Phase 2. Since these two positions were peripheral, participants had to move their eyes away from the center to locate the Y target.

### Preregistered analysis

RTs from Phase 1B were analyzed with a 2 (configuration: repeated vs. random) ×6 (epoch) analysis of variance (ANOVA). This analysis yielded main effects of configuration, *F*(1, 163) = 585.76, *P* < 0.001, and epoch, *F*(4.61, 751.48) = 11.70, *P* < 0.001, and a significant configuration × epoch interaction, *F*(4.71, 767.30) = 3.39, *P* = 0.006.

**Figure 4:**
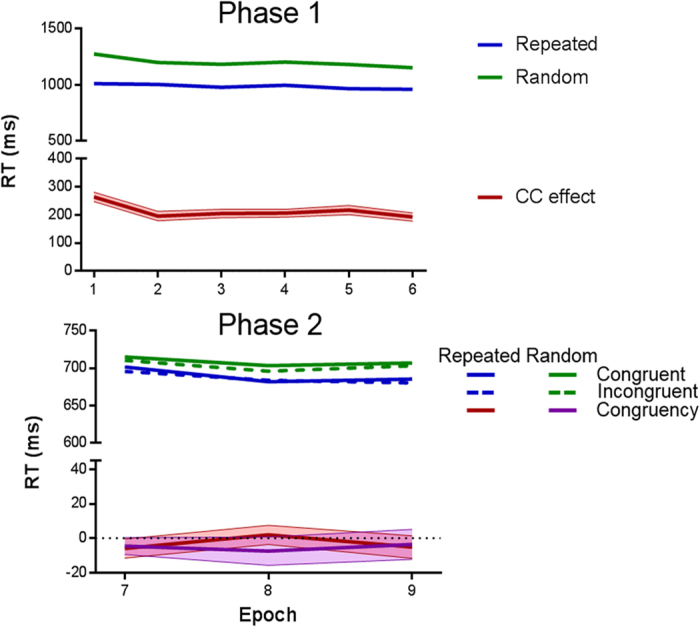
Experiment 3 results. Top panel: Data from Phase 1. ‘CC effect’ refers to the magnitude of the contextual cuing effect (random minus repeated RTs). Bottom panel: Data from Phase 2. ‘Congruency’ refers to the incongruent minus congruent subtraction. Semi-transparent areas depict SEM.

RTs from Stage 2 were analyzed with a 2 (configuration) ×2 (congruency) ×3 (epoch) ANOVA. This analysis yielded a main effect of configuration, *F*(1, 163) = 68.26, *P* < 0.001 (faster RTs for repeated than random configurations), and a marginally significant main effect of epoch, *F*(1.35, 220.42) = 3.29, *P* = 0.058. All remaining main effects and interactions were nonsignificant (smallest *P* > 0.2). The configuration main effect reveals that participants were able to use configurational information in order to speed up the visual search for the new target on repeated trials. However, an inspection of RTs displayed in Fig. 4 suggests that the use of configurational information did not increase the congruency effect whatsoever. In order to confirm this impression, we next performed follow-up analyses to test whether the congruency effect (congruent vs incongruent trials) was significant for either the repeated or random configurations (second phase) – that is collapsing across the epoch variable. For this, we conducted two repeated-measures *t*-tests. These tests did not yield significant results either for repeated, *t*(163) = −0.85, *P* = 0.395, or for random configurations: *t*(163) = −1.11, *P* = 0.268. The congruency effect (which was not significant and numerically the opposite of interference) was not significantly larger for repeated (*M* = −3 ms) than for random (*M* = −5.16 ms) configurations.

The preregistered analysis also included the correlation between the magnitudes of the CC and selective congruency effects, as computed in Experiments 1 and 2, and also specified that this analysis would be 1-tailed. This yielded a null result, *r*(162) = 0.046, *p* > 0.2. The same analysis, but using just the first epoch to compute the size of the selective congruency effect, was also nonsignificant, *r*(162) = 0.012, *p* > 0.4.

### Non-preregistered exploratory analyses

We also performed a Bayesian analysis to measure the magnitude of the null result for the 2 (display) ×2 (congruency) model, as in Experiments 1 and 2. The Bayesian paired *t*-test yielded a BF_01_ = 7.57, substantial evidence for the null hypothesis. The result did not change when we repeated the analysis for the subset of participants who showed CC during Phase 1 (N = 160), BF_01_ = 8.95. The same analysis was repeated for the subset of participants who showed numerical CC in the final epoch of Phase 1 (N = 140), yielding a result even more favorable for the null hypothesis, BF_01_ = 12.66.

In addition, to further explore the main effect of configuration in the second phase, we calculated the correlation between the size of this effect (collapsing across blocks, random minus repeated) and the size of CC in Phase 1B. This correlation was significant, *r*(162) = 0.163, *P* = 0.038 (2-tailed). Hence, the Y was located faster in the repeated patterns during Phase 2, and this effect was positively correlated with the amount of learning produced during the first phase. This positive correlation suggests that the main effect of configuration was not completely produced by new learning during Phase 2, but also by the use of the configurational information acquired for repeated patterns during Phase 1B.

## Discussion

When Dr. Watson claimed that what Holmes sees in a crime scene is “*quite invisible*” to everyone else, Holmes replied: “Not invisible, but unnoticed, Watson. You did not know where to look, and so you missed all that was important.”^15^. Holmes’ attentional orientation to details was (in psychological parlance) the consequence of an effortful, controlled bias voluntarily triggered by the detective pursuing his task goals, which is quite different from the automatic process often assumed to be responsible for CC^4^. However, the results of the current experiments suggest that when context guides visual search we act quite as Holmes did, that is, voluntarily orienting our attention towards those locations which we know are important.

In three experiments we assessed the interference of previously learned CC information on a subsequent phase in which participants controlled their visual orientation looking for targets at already-known relevant positions. If the cuing effect from Phase 1 exerts an uncontrollable influence on Phase 2 performance, our results should show a congruency (i.e., facilitation or interference) effect in repeated patterns but not in random patterns: That is, a configuration (repeated vs random) × congruency (congruent vs incongruent) interaction. If CC is an uncontrollable process, RTs to congruent trials should have been faster than to incongruent trials in the repeated patterns condition where visual search is effectively cued by training in Phase 1. A smaller congruency effect would be expected in random patterns, where no contextual guidance of attention to the T target is anticipated. In random patterns, any congruency effects could only be a consequence of normal unlearned tendencies for attention to spread beyond the expected target (Y) location^16^.

However, across three experiments, involving large samples and addressing a variety of eccentricities, participants effectively suppressed attentional bias towards the T stimulus when they obtained an advantage in doing so. It could be argued that this result was produced simply because perceiving the T stimulus did not affect responding to the Y stimulus. Experiments 1 and 2 ruled out this alternative explanation. The T stimulus’ orientation affected responses to Y as expected, but only (in Experiment 2) when both stimuli were presented in foveal vision (+1 condition). This congruency effect became smaller as the T stimulus was presented farther from the target Y stimulus. Crucially, this effect was similar in repeated and in random displays. Still, it could be argued that because the congruency effect in the random displays (i.e., our baseline) was numerically small, sensitivity for detecting a configuration × congruency interaction was correspondingly low. However, a relatively small baseline is not an issue for two reasons: first, because we predicted *larger* congruency effects for repeated than random displays, and secondly, because despite their small magnitude, our procedures were sufficiently sensitive to detect them. Indeed, we found main effects of congruency in Experiments 1 and 2, and an interaction involving the congruency effect in Experiment 2. Thus, it is reasonable to assume that any CC influence on the congruency effect would also be detectable.

Controlled processes may in some circumstances permit individuals to suppress the activation of automatic mechanisms^17^. It might be possible that participants in Experiments 1 and 2 were so good at focusing attention at the center of the screen that they actually did not perceive the parafoveal L distractors which constitute the repeated configurations. Thus, because uncontrollable orientation towards the T stimulus is assumed to be triggered by the perception of these distractors, it would be reasonable not to obtain it because the configuration of distractors was not perceived. However, this account cannot explain Experiment 3’s main effect of configuration. In the second phase the position of the T stimulus (in terms of the right or the left side of the screen) was perfectly correlated with the position of the Y stimulus, and hence participants could infer where the Y stimulus would be from the repeated displays, using configurational information learned in Phase 1. It seems that participants indeed retrieved the patterns learned in the first phase, and used them to learn where to attend (left or right) to detect the new target. Perception of the repeated pattern did not trigger any automatic orientation to the previously relevant target T; on the contrary, participants flexibly used this information to improve their performance in the new search task. This kind of flexible use of information is not what would be expected from an automatic process, and it supports the idea that CC is likely produced by controlled attention.

The current results could be explained straightforwardly if we assume that the CC effect is not a consequence of more efficient visual search, but a facilitation in response-related processes (e.g. ref. 18). However, many recent studies have established beyond dispute that CC involves enhanced efficiency of visual search (e.g., fewer saccades and fixations) for repeated configurations (see ref. 3), and current debate is more concerned with whether CC relies on orientation guided by the global configuration, or solely by distractors in the local area around the target. Therefore, it might be the case that our participants just learned about the distractors proximal to the T target during Phase 1. Then, in Phase 2 the Y target was too far away from the local area around the T target to be affected by automatic attentional guidance drawn to the T by the distractors close to it. But, as we explained above, participants actually used the knowledge acquired during Phase 1—regardless of whether it was global or local—for improving search efficiency during Phase 2 in Experiment 3, and still no evidence of automatic orientation was found. In addition, since participants clearly perceived the T target in the second phase of Experiments 1 and 2 (as revealed by the significant congruency effects), it is reasonable to assume that they also perceived the distractors around the target—and therefore that automatic attentional orientation towards the target would be still expected.

As is the case with any limited set of observations, the current results have to be interpreted as circumscribed by the particular parametric conditions of our experiments. It is well-known that changes in environmental and/or cognitive factors can facilitate or hinder the operation of controlled mechanisms^19^. For instance, automatic-like responses are more often observed when participants are prompted to respond rapidly, for instance when some time limit for responding is imposed^20^. Thus, it is not unreasonable to think that some automatic attentional orientation might be still observed under conditions less favorable for top-down control processing—e.g., with speeded responses. Future experiments should address that question. However, it is important to note that we selected our task parameters so that they were similar to those typically employed in CC experiments. Thus, we think that is safe to say that the results and conclusion revealed by the current study can be generalized, at least, to the majority of CC studies.

Although the current study is the first one to systematically assess the controllability of CC, some previous literature has addressed this issue in an indirect way. It has been shown that, when the position of the T stimulus is changed in repeated patterns, relearning the new position is hard and requires very extensive training (e.g. refs 21 and 22). This effect is interpreted as resulting from proactive interference from the previous CC regularities and could seem as supportive for the automaticity claim. However, the interference effect vanishes when participants are informed that the position of the target will change^23^. Thus, participants can overrule the proactive interference produced by previous CC learning when they are warned about it. That is the pattern of results that would be expected from a controlled process. These results, jointly with the current data, are compatible with the hypothesis that contextual cuing of visual search is not supported by uncontrollable attentional orientation towards the target in repeated trials. This idea will benefit from future research examining the controllability of CC, for instance by using alternative techniques for measuring attentional orientation, such as event-related potentials^24^, fMRI or/and eye-tracking^25^.

## Methods

### Ethical approval

All experimental protocols were approved by the University of Málaga (Spain) and University College London (UK) Human Investigation Committees, and all tests were carried out in accordance with the approved guidelines. All participants signed approved informed consent forms.

### Participants and apparatus

Stimulus presentation and response recording was handled by software programmed using MatLab, Cogent 2000 and Cogent Graphics.

#### Experiment 1

A total of 30 participants, recruited from the UCL subject pool, took part in the experiment in exchange for a monetary reward of £4. The experiment lasted approximately 40 minutes and was conducted on PCs with 19” TFT monitors set at a resolution of 640 × 480.

#### Experiment 2 and 3

A total of 106 and164 psychology students from the University of Málaga took part in Experiment 1 and 2 (respectively) in exchange for course credit. Experiment 2 comprised two sessions of approximately 40 minutes each, while Experiment 3 comprised one session. They were conducted on PCs with 17” CRT monitors set at a resolution of 800 × 600.

### Materials

Distractor stimuli were sixteen letter Ls and the target stimulus was a letter T during Phase 1 and a letter Y during Phase 2. The fixation cross (displayed centrally before each trial) was a 12 mm square black cross and the background color of the screen was grey. Stimuli were colored blue, red, green or yellow. Distractor stimuli were oriented by rotating the letter L by 0°, 90°, 180°, or 270°. The position, color, and orientation of distractors were randomly assigned for each pattern. Target stimuli T and Y were oriented by rotating the letters by 90° or 270°. Repeated elements of patterns maintained the same position, color, and orientation for distractor stimuli across repetitions.

In both phases, within each block, trials were presented in a random order with the constraint that consecutive trials across adjoining blocks could not present the same repeated configuration. Any given target position could not occur on consecutive trials.

#### Experiment 1

The letter stimuli were approximately 10 mm square (for instance, the long and short line of the T-shaped stimuli was 8.4 mm and 6 mm respectively). For the distractors, the vertical line of the letter L was offset slightly (less than 1 mm) from the end of the horizontal line in order to increase the similarity between distractor and target shapes and therefore increase the difficulty of the visual search task. Stimuli were arranged in an invisible rectangular grid of 144 evenly spaced cells (12 × 12) which was positioned centrally on the screen (Fig. 1A).

Target stimuli in Phase 1 could occur in each of four possible locations at the end of the 120-pixels (≈7.5 cm) long arms of an imaginary cross placed at the center of the screen. Target color was randomly assigned. The position, color, and orientation of distractors were randomly assigned with the constraint that four distractor stimuli (one of each color) appeared in each of the four screen quadrants. Target stimuli in Phase 2 were always located in the center of the screen and color was randomly assigned.

Phase 1 consisted of a total of 480 trials, grouped into 30 blocks of 16 trials each. 8 trials of each block included repeated configurations of distractors-target stimulus and 8 trials of non-repeated, random configurations. Patterns consisted of 17 stimuli (16 distractors plus 1 target). 4 different repeated configurations were included. For each repeated configuration, the target stimulus always appeared in the same position. Each of these four repeated configurations was presented twice in each block; once with the letter T rotated 90° and once with a rotation of 270°.

Phase 2 consisted of a total of 320 trials, grouped into 10 blocks of 32 trials each. 16 trials included repeated distractor-target configurations and 16 trials were non-repeated configurations. Patterns now consisted of 18 stimuli as a new target stimulus was added to the Phase 1 patterns. Repeated configurations were as in Phase 1. Each repeated configuration was presented four times per block; two with the letter Y rotated 90° (one of them included the letter T rotated 90° and the other one rotated 270°) and two with the letter Y rotated 270° (one of them included the letter T rotated 90° and the other one rotated 270°). In two of these presentations the orientation of the letters T and Y was the same (i.e., congruent trials) whereas in the other two it was different (i.e., incongruent trials) (Fig. 1B).

#### Experiment 2

Stimuli were marginally larger than in Experiment 1 and the vertical line of the L-shaped distractors was not offset, making the task marginally easier than in Experiment 1. All stimuli (targets and distractors) were positioned in an invisible squared 11 × 11 grid, where the central column and row were reserved only for target locations (Fig. 1A). Cell size was 50 × 50 pixels (≈2.2 cm).

In Experiments 2 and 3 we did not constrain the number of distractors of each color that could appear in each quadrant. In addition, the T- and Y-shaped targets were always of the same color in Experiment 2 on a given trial, although that color could change from trial to trial.

In Experiment 2, the distance of the targets to the center of the screen was manipulated within-participants. Specifically, targets could appear one row/column away from the center (+1), two rows/columns away from the center (+2), or four columns away from the center (+4). Thus, targets in the +1 and +2 conditions appeared closer to the center than in Experiment 1, whereas in the +4 condition they appeared further away. The number of repeated patterns used for each participant increased to 12, making the task substantially longer than the one used in Experiment 1. To avoid fatigue, the experiment was divided into two sessions. All participants completed Phase 1 A one day, and Phases 1B and 2 on the following day. During Phase 1 A participants were only presented with repeated patterns, while during Phase 1B both repeated and random patterns were presented. 48 blocks were used in Phase 1 A, each of them consisting of one presentation of each of the 12 repeated patterns (4 target locations ×3 eccentricities). 12 blocks were used in Phase 1B, each including the 12 repeated patterns and 12 additional random patterns.

Phase 2 consisted of a total of 576 trials, grouped into 6 blocks of 96 trials each. Half of these testing trials included four presentations of the 12 repeated patterns, each of them with a different combination of orientations for the T-shaped and Y-shaped target. Data were processed as in Experiment 1.

#### Experiment 3

Between-target eccentricity was as in the +2 condition from Experiment 2. Y targets could now appear in two different positions, left or right on the horizontal line which passes through the center at a distance of +4. T-shaped targets could appear in one of four locations, each of them in a different quadrant and with a different color (randomly assigned for each participant), and at a distance of one cell from one of the Y target locations. Thus, T targets could appear above or below the position that the Y target could take during the second phase, and this was the case at both sides of the screen. Both targets always appeared at the same side of the screen (right or left) during the second phase. No distractors were presented on the imaginary line between the possible positions for the Y targets and the center (see Fig. 1A).

Experiment 3’s Phase 1 A comprised 48 blocks of 4 trials each (all repeated configurations), Phase 1B comprised 12 blocks of 8 trials, and Phase 2 comprised 6 blocks of 32 trials.

### Procedure

At the start of the experiment, participants read detailed instructions about the visual search task on the computer screen explaining that their task was to respond to the orientation (pointing left or right) of the target. Example displays were presented and participants could practice the correct response for each orientation. Responses to the target stimulus were made with keys z and m on a standard PC keyboard.

Each trial began with a fixation cross presented in the center of the screen for 1000 ms, and then a pattern of stimuli replaced the fixation cross. Immediately after a correct response, the next trial started. After an incorrect response the word ‘ERROR!’ appeared in a red font at the center of the screen for 2000 ms. After that, the next trial started. A rest break was programmed every 160 trials across both phases of the task. Participants could resume the task after pressing the space bar of the keyboard.

Once Phase 1 was completed, a new set of instructions was presented on the computer screen before starting Phase 2. The new instructions explicitly stated that now the target stimulus whose left/right orientation had to be reported was the letter Y and, in Experiments 1 and 2, that it will always appear in the center of the screen: ‘Now you’ll have to respond to the direction of the Y. The Y will be displayed at the center of the screen’. Accordingly, in Experiment 3 this instruction stated the two possible locations of the Y. After reading the instructions, participants pressed the space bar to resume the task and Phase 2 started as described for Phase 1.

### Data preprocessing

RTs were filtered removing data from trials with incorrect responses and also any RT greater than 10 seconds. Then for each participant we calculated the mean RT during Phases 1 and 2 separately. All RTs more than 3 standard deviations above or below the average (for that phase) were removed from the analysis. To reduce noise and improve statistical power, blocks were collapsed into two-block epochs (i.e., Blocks 1 and 2 were collapsed into Epoch 1, and so on).

## Additional Information

**How to cite this article**: Luque, D. *et al*. Testing the controllability of contextual cuing of visual search. *Sci. Rep.* **7**, 39645; doi: 10.1038/srep39645 (2017).

**Publisher's note:** Springer Nature remains neutral with regard to jurisdictional claims in published maps and institutional affiliations.

## References

[b1] Oliva A. & Torralba A. The role of context in object recognition. Trends Cogn Sci. 11, 520–527 (2007). 18024143 10.1016/j.tics.2007.09.009

[b2] Chun M. M. & Jiang Y. Contextual cueing: Implicit learning and memory of visual context guides spatial attention. Cogn Psychol. 36, 28–71 (1998). 9679076 10.1006/cogp.1998.0681

[b3] Goujon A., Didierjean A. & Thorpe S. Investigating implicit statistical learning mechanisms through contextual cuing. Trends Cogn Sci. 19, 524–533 (2015). 26255970 10.1016/j.tics.2015.07.009

[b4] Chun M. M. & Jiang Y. Implicit, long-term spatial contextual memory. J Exp Psychol Learn Mem Cogn. 29, 224–234 (2003). 12696811 10.1037/0278-7393.29.2.224

[b5] Colagiuri B. & Livesey E. Contextual cuing as a form of nonconscious learning: Theoretical and empirical analysis in large and very large samples. Psychon Bull Rev. 23, 1996–2009 (2016). 27220995 10.3758/s13423-016-1063-0

[b6] Vadillo M. A., Konstantinidis E. & Shanks D. R. Underpowered samples, false negatives, and unconscious learning. Psychon Bull Rev. 23, 87–102 (2016). 26122896 10.3758/s13423-015-0892-6 PMC4742512

[b7] Bargh J. The four horsemen of automaticity: intention, awareness, efficiency, and control as separate issues In Handbook of Social Cognition (eds Wyer R. & Srull T.) 1–40. (Mahwah, NJ: Erlbaum, 1994).

[b8] Moors A. & De Houwer J. Automaticity: a theoretical and conceptual analysis. Psychol Bull. 132, 297–326 (2006). 16536645 10.1037/0033-2909.132.2.297

[b9] Travis S. L., Mattingley J. B. & Dux P. E. On the role of working memory in spatial contextual cueing. J Exp Psychol Learn Mem Cogn. 39, 208–219 (2013). 22642237 10.1037/a0028644

[b10] Jonides J. Voluntary versus automatic control over the mind’s eye’s movement In Attention and Performance IX (eds Long J. B. & Baddeley A. D.) 187–203 (Hillsdale, NJ: Erlbaum, 1981).

[b11] Perlman A. & Tzelgov J. Interactions between encoding and retrieval in the domain of sequence-learning. J Exp Psychol Learn Mem Cogn. 32, 118–130 (2006). 16478345 10.1037/0278-7393.32.1.118

[b12] MacLeod C. M. Half a century of research on the Stroop effect: an integrative review. Psychol Bull. 109, 163–203 (1991). 2034749 10.1037/0033-2909.109.2.163

[b13] Wetzels R. . Statistical evidence in experimental psychology: An empirical comparison using 855 t tests. Perspect Psychol Sci. 6, 291–298 (2011). 26168519 10.1177/1745691611406923

[b14] Zellin M., Conci M., von Mühlenen A. & Müller H. J. Here today, gone tomorrow–adaptation to change in memory-guided visual search. PLoS ONE 8, e59466 (2013). 23555038 10.1371/journal.pone.0059466 PMC3598746

[b15] Conan Doyle A. Sherlock Holmes: A Case of Identity. *The Strand Magazine (September* 1891).

[b16] Lachter J., Forster K. I. & Ruthruff E. Forty-five years after Broadbent (1958): Still no identification without attention. Psychol Rev. 111, 880–913 (2004). 15482066 10.1037/0033-295X.111.4.880

[b17] Seger C. A. Implicit learning. Psychol Bull. 115, 163–196 (1994). 8165269 10.1037/0033-2909.115.2.163

[b18] Kunar M. A., Flusberg S., Horowitz T. S. & Wolfe J. M. Does contextual cuing guide the deployment of attention? J Exp Psychol Hum Percept Perform. 33, 816–828 (2007). 17683230 10.1037/0096-1523.33.4.816 PMC2922990

[b19] Sloman S. A. The empirical case for two systems of reasoning. Psychol Bull. 119, 3–22 (1996).

[b20] Cobos P. L., Gutiérrez-Cobo M. J., Morís J. & Luque D. The effect of dependent measure and time constraints on the expression of different and conflicting generalization processes. J Exp Psychol Learn Mem Cogn. Advance online publication, doi: 10.1037/10.1037/xlm0000335. 27841447

[b21] Conci M., Sun L. & Müller H. J. Contextual remapping in visual search after predictable target-location changes. Psychol Res. 75, 279–289 (2011). 20725739 10.1007/s00426-010-0306-3

[b22] Zellin M., von Mühlenen A., Müller H. J. & Conci M. Long-term adaptation to change in implicit contextual learning. Psychon Bull Rev. 21, 1073–1079 (2014). 24395095 10.3758/s13423-013-0568-z

[b23] Conci M. & Müller H. J. Contextual learning of multiple target locations in visual search. Vis Cogn. 20, 746–770 (2012).

[b24] Luque D., Morís J., Rushby J. A. & Le Pelley M. E. Goal‐directed EEG activity evoked by discriminative stimuli in reinforcement learning. Psychophysiology 52, 238–248 (2015). 25098203 10.1111/psyp.12302

[b25] Manelis A. & Reder L. M. Procedural learning and associative memory mechanisms contribute to contextual cueing: Evidence from fMRI and eye-tracking. Learn Mem. 19, 527–534 (2012). 23073642 10.1101/lm.025973.112 PMC3475156

